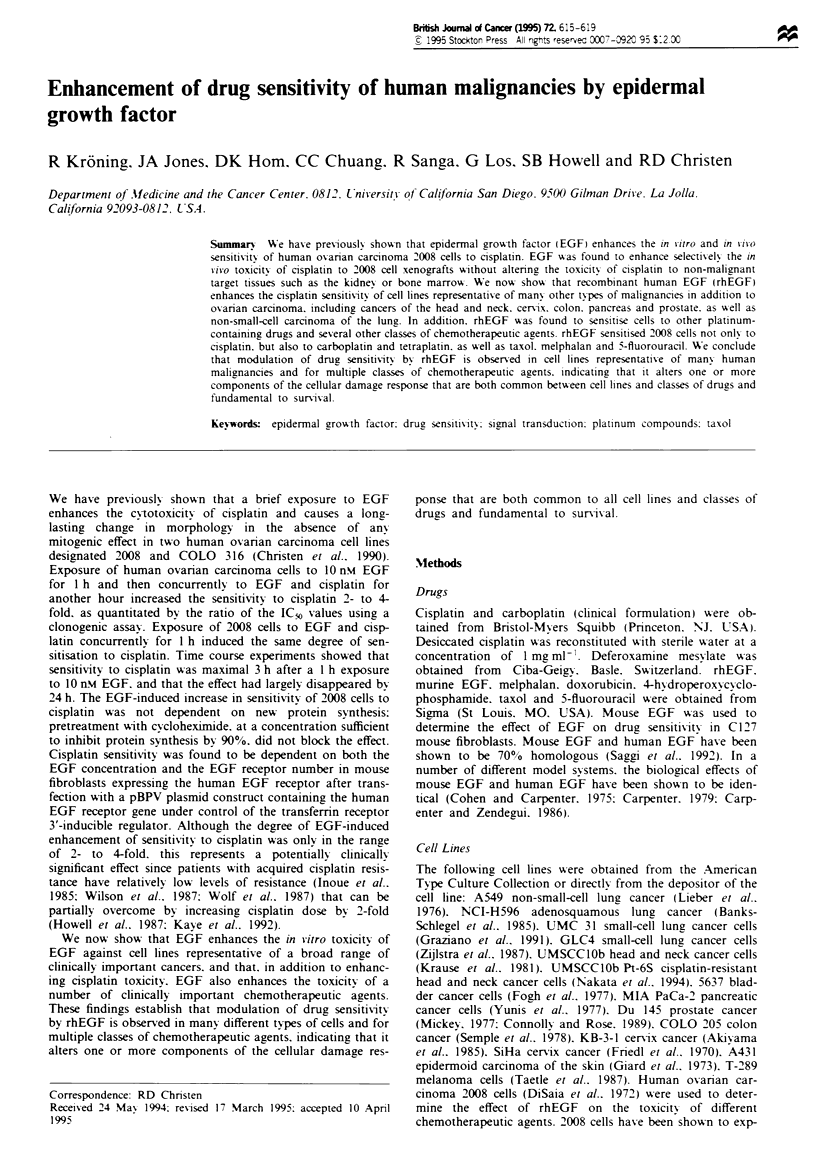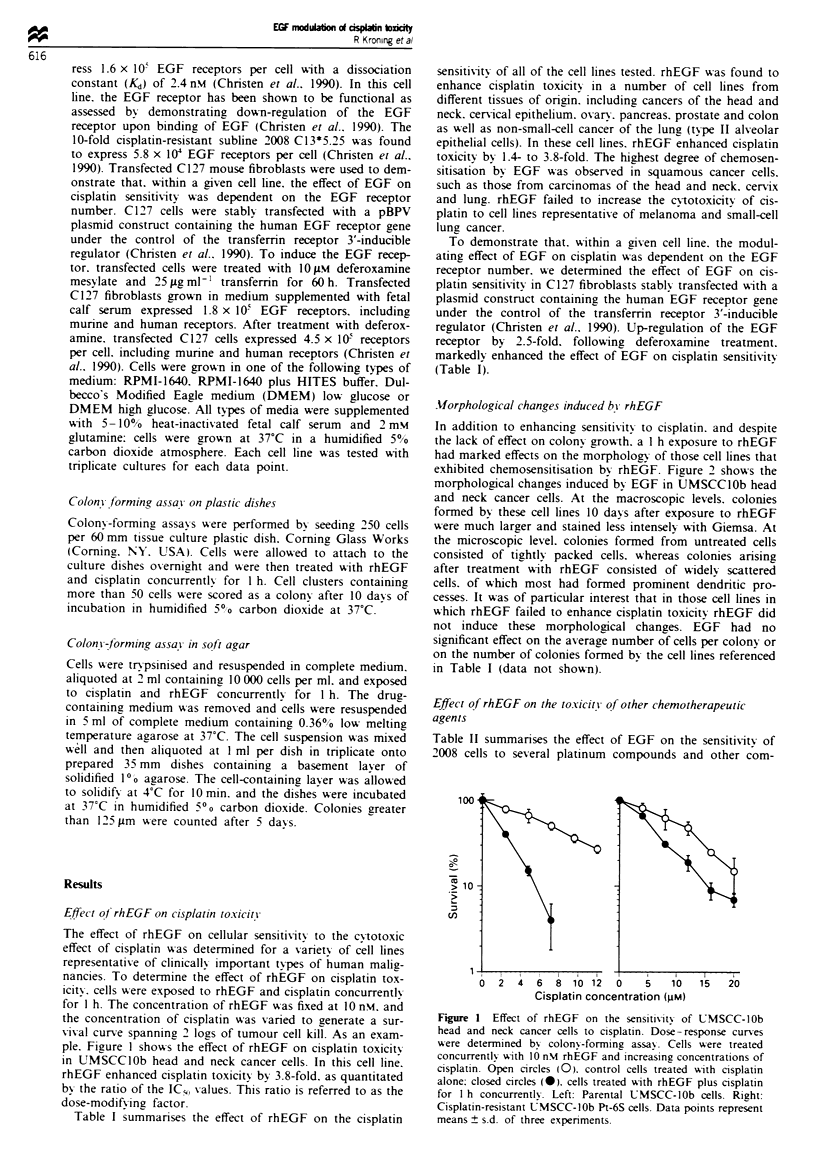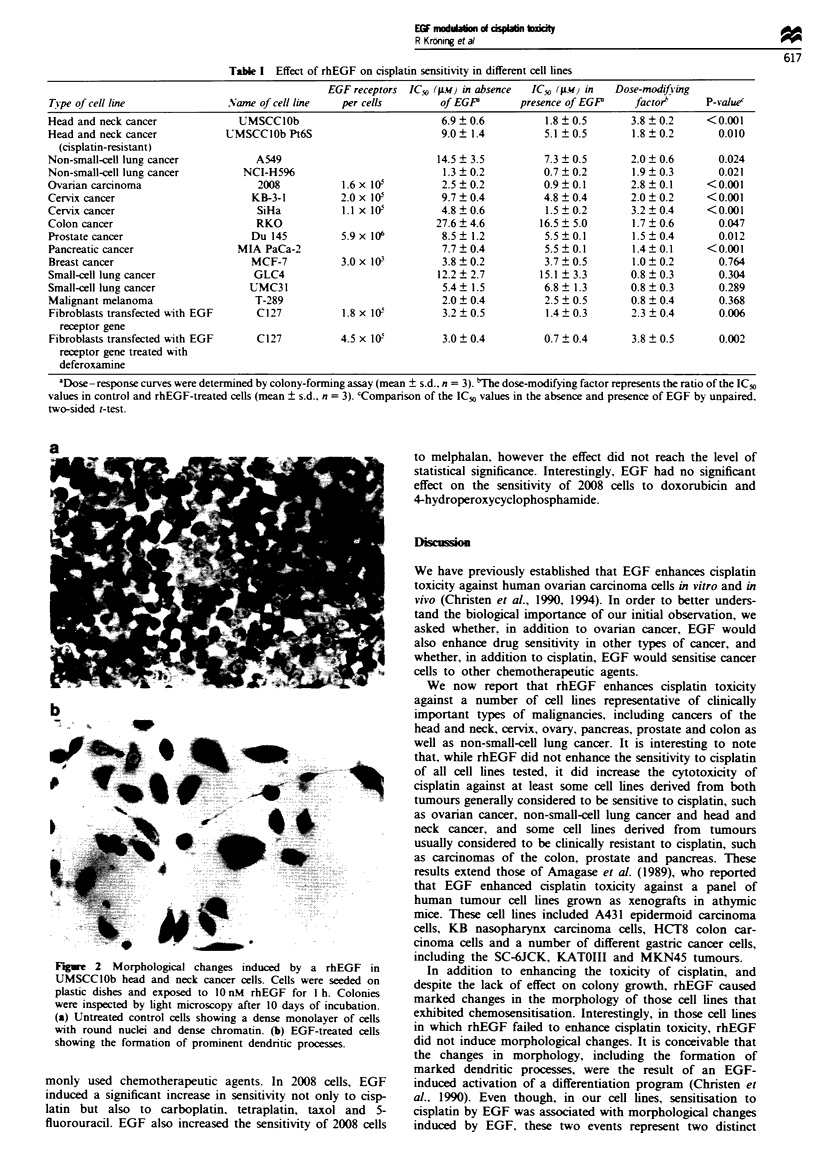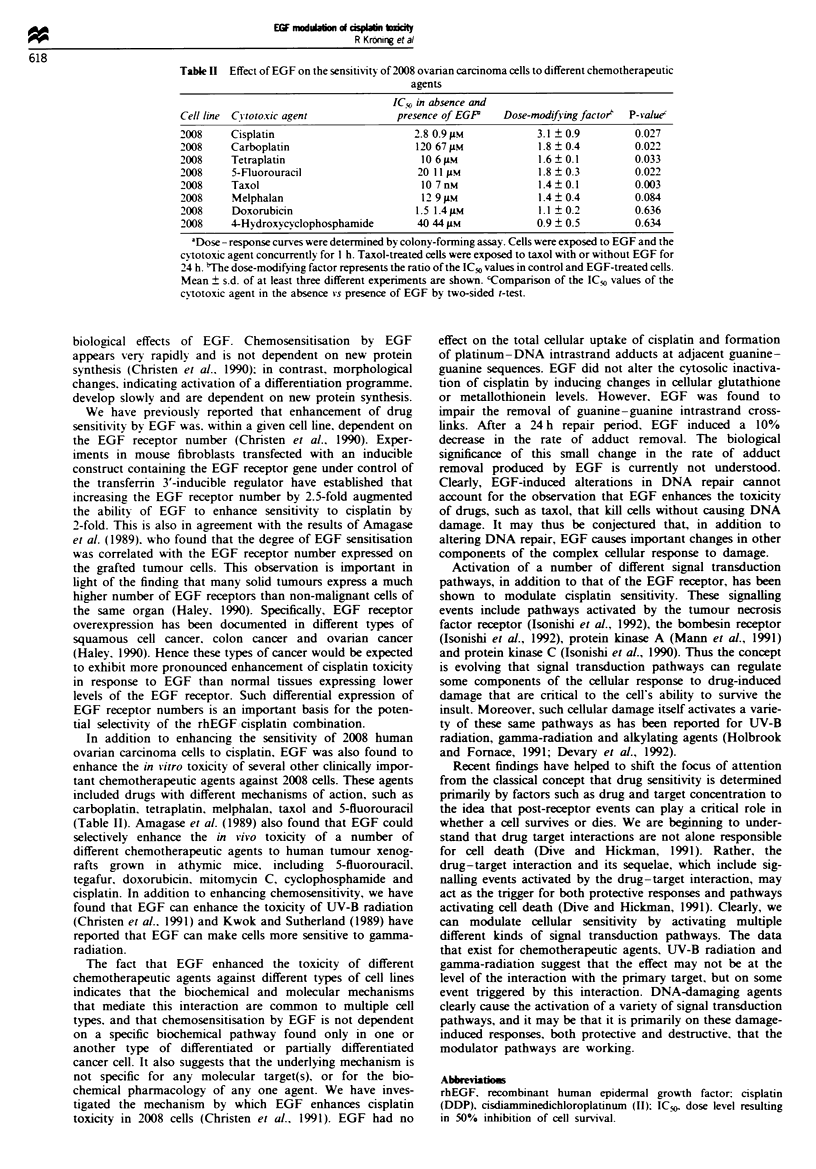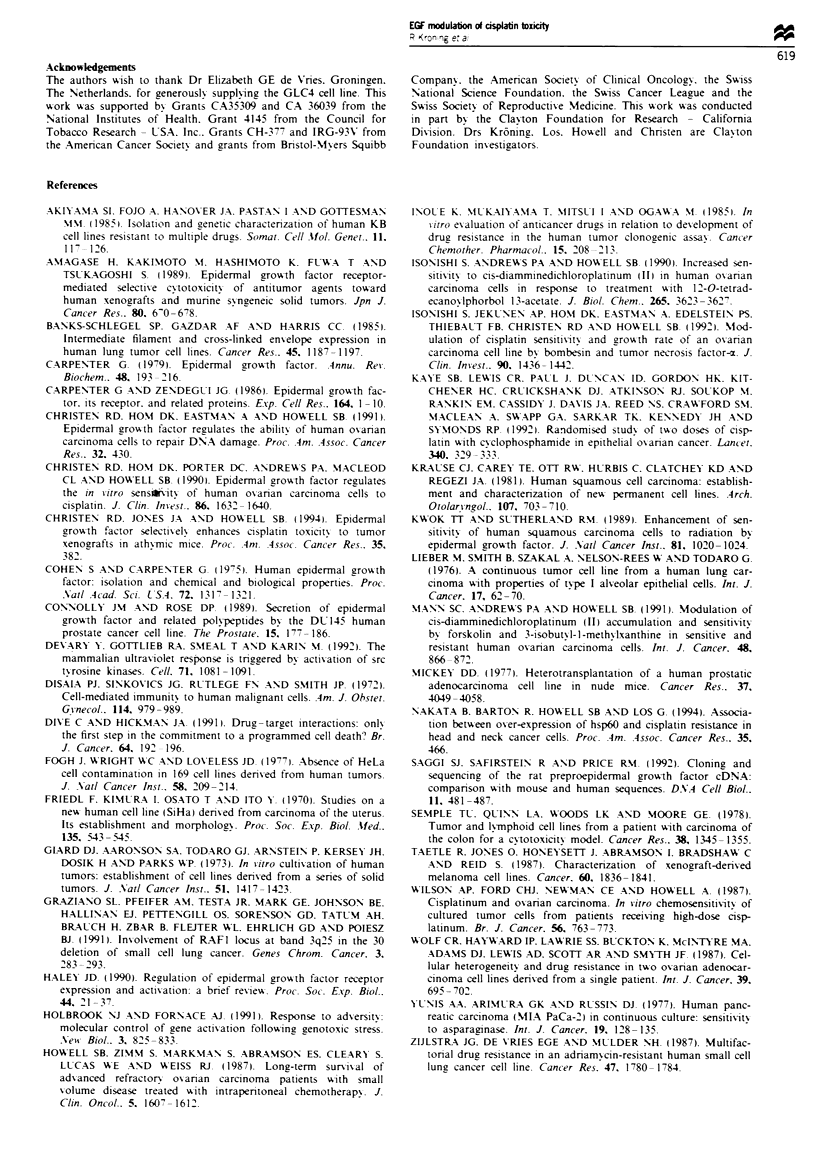# Enhancement of drug sensitivity of human malignancies by epidermal growth factor.

**DOI:** 10.1038/bjc.1995.382

**Published:** 1995-09

**Authors:** R. Kröning, J. A. Jones, D. K. Hom, C. C. Chuang, R. Sanga, G. Los, S. B. Howell, R. D. Christen

**Affiliations:** Department of Medicine, University of California San Diego, La Jolla 92093-0812, USA.

## Abstract

**Images:**


British Journal of Cancer (1995) 72, 615-619

© 1995 Stockton Press All rights reserved 0007-0920/95 $12.00

### Enhancement of drug sensitivity of human malignancies by epidermal growth factor

R Kröning, JA Jones, DK Hom, CC Chuang, R Sanga, G Los, SB Howell and RD Christen

*Department of Medicine and the Cancer Center, 0812, University of California San Diego, 9500 Gilman Drive, La Jolla, California 92093-0812, USA.*

**Summary** We have previously shown that epidermal growth factor (EGF) enhances the *in vitro* and *in vivo* sensitivity of human ovarian carcinoma 2008 cells to cisplatin. EGF was found to enhance selectively the *in vivo* toxicity of cisplatin to 2008 cell xenografts without altering the toxicity of cisplatin to non-malignant target tissues such as the kidney or bone marrow. We now show that recombinant human EGF (rhEGF) enhances the cisplatin sensitivity of cell lines representative of many other types of malignancies in addition to ovarian carcinoma, including cancers of the head and neck, cervix, colon, pancreas and prostate, as well as non-small-cell carcinoma of the lung. In addition, rhEGF was found to sensitise cells to other platinum-containing drugs and several other classes of chemotherapeutic agents. rhEGF sensitised 2008 cells not only to cisplatin, but also to carboplatin and tetraplatin, as well as taxol, melphalan and 5-fluorouracil. We conclude that modulation of drug sensitivity by rhEGF is observed in cell lines representative of many human malignancies and for multiple classes of chemotherapeutic agents, indicating that it alters one or more components of the cellular damage response that are both common between cell lines and classes of drugs and fundamental to survival.

**Keywords:** epidermal growth factor; drug sensitivity; signal transduction; platinum compounds; taxol

We have previously shown that a brief exposure to EGF enhances the cytotoxicity of cisplatin and causes a long-lasting change in morphology in the absence of any mitogenic effect in two human ovarian carcinoma cell lines designated 2008 and COLO 316 (Christen *et al.*, 1990). Exposure of human ovarian carcinoma cells to 10 nM EGF for 1 h and then concurrently to EGF and cisplatin for another hour increased the sensitivity to cisplatin 2- to 4-fold, as quantitated by the ratio of the $IC_{50}$ values using a clonogenic assay. Exposure of 2008 cells to EGF and cisplatin concurrently for 1 h induced the same degree of sensitisation to cisplatin. Time course experiments showed that sensitivity to cisplatin was maximal 3 h after a 1 h exposure to 10 nM EGF, and that the effect had largely disappeared by 24 h. The EGF-induced increase in sensitivity of 2008 cells to cisplatin was not dependent on new protein synthesis: pretreatment with cycloheximide, at a concentration sufficient to inhibit protein synthesis by 90%, did not block the effect. Cisplatin sensitivity was found to be dependent on both the EGF concentration and the EGF receptor number in mouse fibroblasts expressing the human EGF receptor after transfection with a pBPV plasmid construct containing the human EGF receptor gene under control of the transferrin receptor 3'-inducible regulator. Although the degree of EGF-induced enhancement of sensitivity to cisplatin was only in the range of 2- to 4-fold, this represents a potentially clinically significant effect since patients with acquired cisplatin resistance have relatively low levels of resistance (Inoue *et al.*, 1985; Wilson *et al.*, 1987; Wolf *et al.*, 1987) that can be partially overcome by increasing cisplatin dose by 2-fold (Howell *et al.*, 1987; Kaye *et al.*, 1992).

We now show that EGF enhances the *in vitro* toxicity of EGF against cell lines representative of a broad range of clinically important cancers, and that, in addition to enhancing cisplatin toxicity, EGF also enhances the toxicity of a number of clinically important chemotherapeutic agents. These findings establish that modulation of drug sensitivity by rhEGF is observed in many different types of cells and for multiple classes of chemotherapeutic agents, indicating that it alters one or more components of the cellular damage response that are both common to all cell lines and classes of drugs and fundamental to survival.

Correspondence: RD Christen
Received 24 May 1994; revised 17 March 1995; accepted 10 April 1995

### Methods

### *Drugs*

Cisplatin and carboplatin (clinical formulation) were obtained from Bristol-Myers Squibb (Princeton, NJ, USA). Desiccated cisplatin was reconstituted with sterile water at a concentration of 1 mg ml$^{-1}$. Deferoxamine mesylate was obtained from Ciba-Geigy, Basle, Switzerland. rhEGF, murine EGF, melphalan, doxorubicin, 4-hydroperoxycyclophosphamide, taxol and 5-fluorouracil were obtained from Sigma (St Louis, MO, USA). Mouse EGF was used to determine the effect of EGF on drug sensitivity in C127 mouse fibroblasts. Mouse EGF and human EGF have been shown to be 70% homologous (Saggi *et al.*, 1992). In a number of different model systems, the biological effects of mouse EGF and human EGF have been shown to be identical (Cohen and Carpenter, 1975; Carpenter, 1979; Carpenter and Zendegui, 1986).

### *Cell Lines*

The following cell lines were obtained from the American Type Culture Collection or directly from the depositor of the cell line: A549 non-small-cell lung cancer (Lieber *et al.*, 1976), NCI-H596 adenosquamous lung cancer (Banks-Schlegel *et al.*, 1985), UMC 31 small-cell lung cancer cells (Graziano *et al.*, 1991), GLC4 small-cell lung cancer cells (Zijlstra *et al.*, 1987), UMSCC10b head and neck cancer cells (Krause *et al.*, 1981), UMSCC10b Pt-6S cisplatin-resistant head and neck cancer cells (Nakata *et al.*, 1994), 5637 bladder cancer cells (Fogh *et al.*, 1977), MIA PaCa-2 pancreatic cancer cells (Yunis *et al.*, 1977), Du 145 prostate cancer (Mickey, 1977; Connolly and Rose, 1989), COLO 205 colon cancer (Semple *et al.*, 1978), KB-3-1 cervix cancer (Akiyama *et al.*, 1985), SiHa cervix cancer (Friedl *et al.*, 1970), A431 epidermoid carcinoma of the skin (Giard *et al.*, 1973), T-289 melanoma cells (Taetle *et al.*, 1987). Human ovarian carcinoma 2008 cells (DiSaia *et al.*, 1972) were used to determine the effect of rhEGF on the toxicity of different chemotherapeutic agents. 2008 cells have been shown to express $1.6 \times 10^5$ EGF receptors per cell with a dissociation constant ($K_d$) of 2.4 nM (Christen *et al.*, 1990). In this cell line, the EGF receptor has been shown to be functional as assessed by demonstrating down-regulation of the EGF receptor upon binding of EGF (Christen *et al.*, 1990). The 10-fold cisplatin-resistant subline 2008 C13*5.25 was found to express $5.8 \times 10^4$ EGF receptors per cell (Christen *et al.*, 1990). Transfected C127 mouse fibroblasts were used to demonstrate that, within a given cell line, the effect of EGF on cisplatin sensitivity was dependent on the EGF receptor number. C127 cells were stably transfected with a pBPV plasmid construct containing the human EGF receptor gene under the control of the transferrin receptor 3′-inducible regulator (Christen *et al.*, 1990). To induce the EGF receptor, transfected cells were treated with 10 μM deferoxamine mesylate and 25 $\mu g\ ml^{-1}$ transferrin for 60 h. Transfected C127 fibroblasts grown in medium supplemented with fetal calf serum expressed $1.8 \times 10^5$ EGF receptors, including murine and human receptors. After treatment with deferoxamine, transfected C127 cells expressed $4.5 \times 10^5$ receptors per cell, including murine and human receptors (Christen *et al.*, 1990). Cells were grown in one of the following types of medium: RPMI-1640, RPMI-1640 plus HITES buffer, Dulbecco's Modified Eagle medium (DMEM) low glucose or DMEM high glucose. All types of media were supplemented with 5–10% heat-inactivated fetal calf serum and 2 mM glutamine; cells were grown at 37°C in a humidified 5% carbon dioxide atmosphere. Each cell line was tested with triplicate cultures for each data point.

### *Colony forming assay on plastic dishes*

Colony-forming assays were performed by seeding 250 cells per 60 mm tissue culture plastic dish, Corning Glass Works (Corning, NY, USA). Cells were allowed to attach to the culture dishes overnight and were then treated with rhEGF and cisplatin concurrently for 1 h. Cell clusters containing more than 50 cells were scored as a colony after 10 days of incubation in humidified 5% carbon dioxide at 37°C.

### *Colony-forming assay in soft agar*

Cells were trypsinised and resuspended in complete medium, aliquoted at 2 ml containing 10 000 cells per ml, and exposed to cisplatin and rhEGF concurrently for 1 h. The drug-containing medium was removed and cells were resuspended in 5 ml of complete medium containing 0.36% low melting temperature agarose at 37°C. The cell suspension was mixed well and then aliquoted at 1 ml per dish in triplicate onto prepared 35 mm dishes containing a basement layer of solidified 1% agarose. The cell-containing layer was allowed to solidify at 4°C for 10 min, and the dishes were incubated at 37°C in humidified 5% carbon dioxide. Colonies greater than 125 μm were counted after 5 days.

### Results

### *Effect of rhEGF on cisplatin toxicity*

The effect of rhEGF on cellular sensitivity to the cytotoxic effect of cisplatin was determined for a variety of cell lines representative of clinically important types of human malignancies. To determine the effect of rhEGF on cisplatin toxicity, cells were exposed to rhEGF and cisplatin concurrently for 1 h. The concentration of rhEGF was fixed at 10 nM, and the concentration of cisplatin was varied to generate a survival curve spanning 2 logs of tumour cell kill. As an example, Figure 1 shows the effect of rhEGF on cisplatin toxicity in UMSCC10b head and neck cancer cells. In this cell line, rhEGF enhanced cisplatin toxicity by 3.8-fold, as quantitated by the ratio of the $IC_{50}$ values. This ratio is referred to as the dose-modifying factor.

Table I summarises the effect of rhEGF on the cisplatin sensitivity of all of the cell lines tested. rhEGF was found to enhance cisplatin toxicity in a number of cell lines from different tissues of origin, including cancers of the head and neck, cervical epithelium, ovary, pancreas, prostate and colon as well as non-small-cell cancer of the lung (type II alveolar epithelial cells). In these cell lines, rhEGF enhanced cisplatin toxicity by 1.4- to 3.8-fold. The highest degree of chemosensitisation by EGF was observed in squamous cancer cells, such as those from carcinomas of the head and neck, cervix and lung. rhEGF failed to increase the cytotoxicity of cisplatin to cell lines representative of melanoma and small-cell lung cancer.

To demonstrate that, within a given cell line, the modulating effect of EGF on cisplatin was dependent on the EGF receptor number, we determined the effect of EGF on cisplatin sensitivity in C127 fibroblasts stably transfected with a plasmid construct containing the human EGF receptor gene under the control of the transferrin receptor 3′-inducible regulator (Christen *et al.*, 1990). Up-regulation of the EGF receptor by 2.5-fold, following deferoxamine treatment, markedly enhanced the effect of EGF on cisplatin sensitivity (Table I).

### *Morphological changes induced by rhEGF*

In addition to enhancing sensitivity to cisplatin, and despite the lack of effect on colony growth, a 1 h exposure to rhEGF had marked effects on the morphology of those cell lines that exhibited chemosensitisation by rhEGF. Figure 2 shows the morphological changes induced by EGF in UMSCC10b head and neck cancer cells. At the macroscopic levels, colonies formed by these cell lines 10 days after exposure to rhEGF were much larger and stained less intensely with Giemsa. At the microscopic level, colonies formed from untreated cells consisted of tightly packed cells, whereas colonies arising after treatment with rhEGF consisted of widely scattered cells, of which most had formed prominent dendritic processes. It was of particular interest that in those cell lines in which rhEGF failed to enhance cisplatin toxicity rhEGF did not induce these morphological changes. EGF had no significant effect on the average number of cells per colony or on the number of colonies formed by the cell lines referenced in Table I (data not shown).

### *Effect of rhEGF on the toxicity of other chemotherapeutic agents*

Table II summarises the effect of EGF on the sensitivity of 2008 cells to several platinum compounds and other com-

**Figure 1** Effect of rhEGF on the sensitivity of UMSCC-10b head and neck cancer cells to cisplatin. Dose-response curves were determined by colony-forming assay. Cells were treated concurrently with 10 nM rhEGF and increasing concentrations of cisplatin. Open circles (○), control cells treated with cisplatin alone; closed circles (●), cells treated with rhEGF plus cisplatin for 1 h concurrently. Left: Parental UMSCC-10b cells. Right: Cisplatin-resistant UMSCC-10b Pt-6S cells. Data points represent means ± s.d. of three experiments.

Table I Effect of rhEGF on cisplatin sensitivity in different cell lines

| Type of cell line | Name of cell line | EGF receptors per cells | $IC_{50}$ (μM) in absence of EGF[a] | $IC_{50}$ (μM) in presence of EGF[a] | Dose-modifying factor[b] | P-value[c] |
|---|---|---|---|---|---|---|
| Head and neck cancer | UMSCC10b | | 6.9 ± 0.6 | 1.8 ± 0.5 | 3.8 ± 0.2 | <0.001 |
| Head and neck cancer (cisplatin-resistant) | UMSCC10b Pt6S | | 9.0 ± 1.4 | 5.1 ± 0.5 | 1.8 ± 0.2 | 0.010 |
| Non-small-cell lung cancer | A549 | | 14.5 ± 3.5 | 7.3 ± 0.5 | 2.0 ± 0.6 | 0.024 |
| Non-small-cell lung cancer | NCI-H596 | | 1.3 ± 0.2 | 0.7 ± 0.2 | 1.9 ± 0.3 | 0.021 |
| Ovarian carcinoma | 2008 | $1.6 \times 10^5$ | 2.5 ± 0.2 | 0.9 ± 0.1 | 2.8 ± 0.1 | <0.001 |
| Cervix cancer | KB-3-1 | $2.0 \times 10^5$ | 9.7 ± 0.4 | 4.8 ± 0.4 | 2.0 ± 0.2 | <0.001 |
| Cervix cancer | SiHa | $1.1 \times 10^5$ | 4.8 ± 0.6 | 1.5 ± 0.2 | 3.2 ± 0.4 | <0.001 |
| Colon cancer | RKO | | 27.6 ± 4.6 | 16.5 ± 5.0 | 1.7 ± 0.6 | 0.047 |
| Prostate cancer | Du 145 | $5.9 \times 10^6$ | 8.5 ± 1.2 | 5.5 ± 0.1 | 1.5 ± 0.4 | 0.012 |
| Pancreatic cancer | MIA PaCa-2 | | 7.7 ± 0.4 | 5.5 ± 0.1 | 1.4 ± 0.1 | <0.001 |
| Breast cancer | MCF-7 | $3.0 \times 10^3$ | 3.8 ± 0.2 | 3.7 ± 0.5 | 1.0 ± 0.2 | 0.764 |
| Small-cell lung cancer | GLC4 | | 12.2 ± 2.7 | 15.1 ± 3.3 | 0.8 ± 0.3 | 0.304 |
| Small-cell lung cancer | UMC31 | | 5.4 ± 1.5 | 6.8 ± 1.3 | 0.8 ± 0.3 | 0.289 |
| Malignant melanoma | T-289 | | 2.0 ± 0.4 | 2.5 ± 0.5 | 0.8 ± 0.4 | 0.368 |
| Fibroblasts transfected with EGF receptor gene | C127 | $1.8 \times 10^5$ | 3.2 ± 0.5 | 1.4 ± 0.3 | 2.3 ± 0.4 | 0.006 |
| Fibroblasts transfected with EGF receptor gene treated with deferoxamine | C127 | $4.5 \times 10^5$ | 3.0 ± 0.4 | 0.7 ± 0.4 | 3.8 ± 0.5 | 0.002 |

[a]Dose-response curves were determined by colony-forming assay (mean ± s.d., $n = 3$). [b]The dose-modifying factor represents the ratio of the $IC_{50}$ values in control and rhEGF-treated cells (mean ± s.d., $n = 3$). [c]Comparison of the $IC_{50}$ values in the absence and presence of EGF by unpaired, two-sided $t$-test.

**Figure 2** Morphological changes induced by a rhEGF in UMSCC10b head and neck cancer cells. Cells were seeded on plastic dishes and exposed to 10 nM rhEGF for 1 h. Colonies were inspected by light microscopy after 10 days of incubation. (**a**) Untreated control cells showing a dense monolayer of cells with round nuclei and dense chromatin. (**b**) EGF-treated cells showing the formation of prominent dendritic processes.

monly used chemotherapeutic agents. In 2008 cells, EGF induced a significant increase in sensitivity not only to cisplatin but also to carboplatin, tetraplatin, taxol and 5-fluorouracil. EGF also increased the sensitivity of 2008 cells to melphalan, however the effect did not reach the level of statistical significance. Interestingly, EGF had no significant effect on the sensitivity of 2008 cells to doxorubicin and 4-hydroperoxycyclophosphamide.

### Discussion

We have previously established that EGF enhances cisplatin toxicity against human ovarian carcinoma cells *in vitro* and *in vivo* (Christen *et al.*, 1990, 1994). In order to better understand the biological importance of our initial observation, we asked whether, in addition to ovarian cancer, EGF would also enhance drug sensitivity in other types of cancer, and whether, in addition to cisplatin, EGF would sensitise cancer cells to other chemotherapeutic agents.

We now report that rhEGF enhances cisplatin toxicity against a number of cell lines representative of clinically important types of malignancies, including cancers of the head and neck, cervix, ovary, pancreas, prostate and colon as well as non-small-cell lung cancer. It is interesting to note that, while rhEGF did not enhance the sensitivity to cisplatin of all cell lines tested, it did increase the cytotoxicity of cisplatin against at least some cell lines derived from both tumours generally considered to be sensitive to cisplatin, such as ovarian cancer, non-small-cell lung cancer and head and neck cancer, and some cell lines derived from tumours usually considered to be clinically resistant to cisplatin, such as carcinomas of the colon, prostate and pancreas. These results extend those of Amagase *et al.* (1989), who reported that EGF enhanced cisplatin toxicity against a panel of human tumour cell lines grown as xenografts in athymic mice. These cell lines included A431 epidermoid carcinoma cells, KB nasopharynx carcinoma cells, HCT8 colon carcinoma cells and a number of different gastric cancer cells, including the SC-6JCK, KATOIII and MKN45 tumours.

In addition to enhancing the toxicity of cisplatin, and despite the lack of effect on colony growth, rhEGF caused marked changes in the morphology of those cell lines that exhibited chemosensitisation. Interestingly, in those cell lines in which rhEGF failed to enhance cisplatin toxicity, rhEGF did not induce morphological changes. It is conceivable that the changes in morphology, including the formation of marked dendritic processes, were the result of an EGF-induced activation of a differentiation program (Christen *et al.*, 1990). Even though, in our cell lines, sensitisation to cisplatin by EGF was associated with morphological changes induced by EGF, these two events represent two distinct

**Table II** Effect of EGF on the sensitivity of 2008 ovarian carcinoma cells to different chemotherapeutic agents

| Cell line | Cytotoxic agent | $IC_{50}$ in absence and presence of EGF[a] | Dose-modifying factor[b] | P-value[c] |
|---|---|---|---|---|
| 2008 | Cisplatin | 2.8 0.9 μM | 3.1 ± 0.9 | 0.027 |
| 2008 | Carboplatin | 120 67 μM | 1.8 ± 0.4 | 0.022 |
| 2008 | Tetraplatin | 10 6 μM | 1.6 ± 0.1 | 0.033 |
| 2008 | 5-Fluorouracil | 20 11 μM | 1.8 ± 0.3 | 0.022 |
| 2008 | Taxol | 10 7 nM | 1.4 ± 0.1 | 0.003 |
| 2008 | Melphalan | 12 9 μM | 1.4 ± 0.4 | 0.084 |
| 2008 | Doxorubicin | 1.5 1.4 μM | 1.1 ± 0.2 | 0.636 |
| 2008 | 4-Hydroxycyclophosphamide | 40 44 μM | 0.9 ± 0.5 | 0.634 |

[a]Dose–response curves were determined by colony-forming assay. Cells were exposed to EGF and the cytotoxic agent concurrently for 1 h. Taxol-treated cells were exposed to taxol with or without EGF for 24 h. [b]The dose-modifying factor represents the ratio of the $IC_{50}$ values in control and EGF-treated cells. Mean ± s.d. of at least three different experiments are shown. [c]Comparison of the $IC_{50}$ values of the cytotoxic agent in the absence *vs* presence of EGF by two-sided *t*-test.

biological effects of EGF. Chemosensitisation by EGF appears very rapidly and is not dependent on new protein synthesis (Christen *et al.*, 1990); in contrast, morphological changes, indicating activation of a differentiation programme, develop slowly and are dependent on new protein synthesis.

We have previously reported that enhancement of drug sensitivity by EGF was, within a given cell line, dependent on the EGF receptor number (Christen *et al.*, 1990). Experiments in mouse fibroblasts transfected with an inducible construct containing the EGF receptor gene under control of the transferrin 3′-inducible regulator have established that increasing the EGF receptor number by 2.5-fold augmented the ability of EGF to enhance sensitivity to cisplatin by 2-fold. This is also in agreement with the results of Amagase *et al.* (1989), who found that the degree of EGF sensitisation was correlated with the EGF receptor number expressed on the grafted tumour cells. This observation is important in light of the finding that many solid tumours express a much higher number of EGF receptors than non-malignant cells of the same organ (Haley, 1990). Specifically, EGF receptor overexpression has been documented in different types of squamous cell cancer, colon cancer and ovarian cancer (Haley, 1990). Hence these types of cancer would be expected to exhibit more pronounced enhancement of cisplatin toxicity in response to EGF than normal tissues expressing lower levels of the EGF receptor. Such differential expression of EGF receptor numbers is an important basis for the potential selectivity of the rhEGF/cisplatin combination.

In addition to enhancing the sensitivity of 2008 human ovarian carcinoma cells to cisplatin, EGF was also found to enhance the *in vitro* toxicity of several other clinically important chemotherapeutic agents against 2008 cells. These agents included drugs with different mechanisms of action, such as carboplatin, tetraplatin, melphalan, taxol and 5-fluorouracil (Table II). Amagase *et al.* (1989) also found that EGF could selectively enhance the *in vivo* toxicity of a number of different chemotherapeutic agents to human tumour xenografts grown in athymic mice, including 5-fluorouracil, tegafur, doxorubicin, mitomycin C, cyclophosphamide and cisplatin. In addition to enhancing chemosensitivity, we have found that EGF can enhance the toxicity of UV-B radiation (Christen *et al.*, 1991) and Kwok and Sutherland (1989) have reported that EGF can make cells more sensitive to gamma-radiation.

The fact that EGF enhanced the toxicity of different chemotherapeutic agents against different types of cell lines indicates that the biochemical and molecular mechanisms that mediate this interaction are common to multiple cell types, and that chemosensitisation by EGF is not dependent on a specific biochemical pathway found only in one or another type of differentiated or partially differentiated cancer cell. It also suggests that the underlying mechanism is not specific for any molecular target(s), or for the biochemical pharmacology of any one agent. We have investigated the mechanism by which EGF enhances cisplatin toxicity in 2008 cells (Christen *et al.*, 1991). EGF had no effect on the total cellular uptake of cisplatin and formation of platinum–DNA intrastrand adducts at adjacent guanine–guanine sequences. EGF did not alter the cytosolic inactivation of cisplatin by inducing changes in cellular glutathione or metallothionein levels. However, EGF was found to impair the removal of guanine–guanine intrastrand cross-links. After a 24 h repair period, EGF induced a 10% decrease in the rate of adduct removal. The biological significance of this small change in the rate of adduct removal produced by EGF is currently not understood. Clearly, EGF-induced alterations in DNA repair cannot account for the observation that EGF enhances the toxicity of drugs, such as taxol, that kill cells without causing DNA damage. It may thus be conjectured that, in addition to altering DNA repair, EGF causes important changes in other components of the complex cellular response to damage.

Activation of a number of different signal transduction pathways, in addition to that of the EGF receptor, has been shown to modulate cisplatin sensitivity. These signalling events include pathways activated by the tumour necrosis factor receptor (Isonishi *et al.*, 1992), the bombesin receptor (Isonishi *et al.*, 1992), protein kinase A (Mann *et al.*, 1991) and protein kinase C (Isonishi *et al.*, 1990). Thus the concept is evolving that signal transduction pathways can regulate some components of the cellular response to drug-induced damage that are critical to the cell's ability to survive the insult. Moreover, such cellular damage itself activates a variety of these same pathways as has been reported for UV-B radiation, gamma-radiation and alkylating agents (Holbrook and Fornace, 1991; Devary *et al.*, 1992).

Recent findings have helped to shift the focus of attention from the classical concept that drug sensitivity is determined primarily by factors such as drug and target concentration to the idea that post-receptor events can play a critical role in whether a cell survives or dies. We are beginning to understand that drug target interactions are not alone responsible for cell death (Dive and Hickman, 1991). Rather, the drug–target interaction and its sequelae, which include signalling events activated by the drug–target interaction, may act as the trigger for both protective responses and pathways activating cell death (Dive and Hickman, 1991). Clearly, we can modulate cellular sensitivity by activating multiple different kinds of signal transduction pathways. The data that exist for chemotherapeutic agents, UV-B radiation and gamma-radiation suggest that the effect may not be at the level of the interaction with the primary target, but on some event triggered by this interaction. DNA-damaging agents clearly cause the activation of a variety of signal transduction pathways, and it may be that it is primarily on these damage-induced responses, both protective and destructive, that the modulator pathways are working.

**Abbreviations**

rhEGF, recombinant human epidermal growth factor; cisplatin (DDP), cisdiamminedichloroplatinum (II); $IC_{50}$, dose level resulting in 50% inhibition of cell survival.

**Acknowledgements**

The authors wish to thank Dr Elizabeth GE de Vries, Groningen, The Netherlands, for generously supplying the GLC4 cell line. This work was supported by Grants CA35309 and CA 36039 from the National Institutes of Health, Grant 4145 from the Council for Tobacco Research – USA, Inc., Grants CH-377 and IRG-93V from the American Cancer Society and grants from Bristol-Myers Squibb Company, the American Society of Clinical Oncology, the Swiss National Science Foundation, the Swiss Cancer League and the Swiss Society of Reproductive Medicine. This work was conducted in part by the Clayton Foundation for Research – California Division. Drs Kröning, Los, Howell and Christen are Clayton Foundation investigators.

### References

AKIYAMA SI, FOJO A, HANOVER JA, PASTAN I AND GOTTESMAN MM. (1985). Isolation and genetic characterization of human KB cell lines resistant to multiple drugs. *Somat. Cell Mol. Genet.*, **11**, 117–126.

AMAGASE H, KAKIMOTO M, HASHIMOTO K, FUWA T AND TSUKAGOSHI S. (1989). Epidermal growth factor receptor-mediated selective cytotoxicity of antitumor agents toward human xenografts and murine syngeneic solid tumors. *Jpn J. Cancer Res.*, **80**, 670–678.

BANKS-SCHLEGEL SP, GAZDAR AF AND HARRIS CC. (1985). Intermediate filament and cross-linked envelope expression in human lung tumor cell lines. *Cancer Res.*, **45**, 1187–1197.

CARPENTER G. (1979). Epidermal growth factor. *Annu. Rev. Biochem.*, **48**, 193–216.

CARPENTER G AND ZENDEGUI JG. (1986). Epidermal growth factor, its receptor, and related proteins. *Exp. Cell Res.*, **164**, 1–10.

CHRISTEN RD, HOM DK, EASTMAN A AND HOWELL SB. (1991). Epidermal growth factor regulates the ability of human ovarian carcinoma cells to repair DNA damage. *Proc. Am. Assoc. Cancer Res.*, **32**, 430.

CHRISTEN RD, HOM DK, PORTER DC, ANDREWS PA, MACLEOD CL AND HOWELL SB. (1990). Epidermal growth factor regulates the *in vitro* sensitivity of human ovarian carcinoma cells to cisplatin. *J. Clin. Invest.*, **86**, 1632–1640.

CHRISTEN RD, JONES JA AND HOWELL SB. (1994). Epidermal growth factor selectively enhances cisplatin toxicity to tumor xenografts in athymic mice. *Proc. Am. Assoc. Cancer Res.*, **35**, 382.

COHEN S AND CARPENTER G. (1975). Human epidermal growth factor: isolation and chemical and biological properties. *Proc. Natl Acad. Sci. USA*, **72**, 1317–1321.

CONNOLLY JM AND ROSE DP. (1989). Secretion of epidermal growth factor and related polypeptides by the DU145 human prostate cancer cell line. *The Prostate*, **15**, 177–186.

DEVARY Y, GOTTLIEB RA, SMEAL T AND KARIN M. (1992). The mammalian ultraviolet response is triggered by activation of src tyrosine kinases. *Cell*, **71**, 1081–1091.

DISAIA PJ, SINKOVICS JG, RUTLEGE FN AND SMITH JP. (1972). Cell-mediated immunity to human malignant cells. *Am. J. Obstet. Gynecol.*, **114**, 979–989.

DIVE C AND HICKMAN JA. (1991). Drug–target interactions: only the first step in the commitment to a programmed cell death? *Br. J. Cancer*, **64**, 192–196.

FOGH J, WRIGHT WC AND LOVELESS JD. (1977). Absence of HeLa cell contamination in 169 cell lines derived from human tumors. *J. Natl Cancer Inst.*, **58**, 209–214.

FRIEDL F, KIMURA I, OSATO T AND ITO Y. (1970). Studies on a new human cell line (SiHa) derived from carcinoma of the uterus. Its establishment and morphology. *Proc. Soc. Exp. Biol. Med.*, **135**, 543–545.

GIARD DJ, AARONSON SA, TODARO GJ, ARNSTEIN P, KERSEY JH, DOSIK H AND PARKS WP. (1973). *In vitro* cultivation of human tumors: establishment of cell lines derived from a series of solid tumors. *J. Natl Cancer Inst.*, **51**, 1417–1423.

GRAZIANO SL, PFEIFER AM, TESTA JR, MARK GE, JOHNSON BE, HALLINAN EJ, PETTENGILL OS, SORENSON GD, TATUM AH, BRAUCH H, ZBAR B, FLEJTER WL, EHRLICH GD AND POIESZ BJ. (1991). Involvement of RAF1 locus at band 3q25 in the 30 deletion of small cell lung cancer. *Genes Chrom. Cancer*, **3**, 283–293.

HALEY JD. (1990). Regulation of epidermal growth factor receptor expression and activation: a brief review. *Proc. Soc. Exp. Biol.*, **44**, 21–37.

HOLBROOK NJ AND FORNACE AJ. (1991). Response to adversity: molecular control of gene activation following genotoxic stress. *New Biol.*, **3**, 825–833.

HOWELL SB, ZIMM S, MARKMAN S, ABRAMSON ES, CLEARY S, LUCAS WE AND WEISS RJ. (1987). Long-term survival of advanced refractory ovarian carcinoma patients with small volume disease treated with intraperitoneal chemotherapy. *J. Clin. Oncol.*, **5**, 1607–1612.

INOUE K, MUKAIYAMA T, MITSUI I AND OGAWA M. (1985). *In vitro* evaluation of anticancer drugs in relation to development of drug resistance in the human tumor clonogenic assay. *Cancer Chemother. Pharmacol.*, **15**, 208–213.

ISONISHI S, ANDREWS PA AND HOWELL SB. (1990). Increased sensitivity to cis-diamminedichloroplatinum (II) in human ovarian carcinoma cells in response to treatment with 12-*O*-tetradecanoylphorbol 13-acetate. *J. Biol. Chem.*, **265**, 3623–3627.

ISONISHI S, JEKUNEN AP, HOM DK, EASTMAN A, EDELSTEIN PS, THIEBAUT FB, CHRISTEN RD AND HOWELL SB. (1992). Modulation of cisplatin sensitivity and growth rate of an ovarian carcinoma cell line by bombesin and tumor necrosis factor-α. *J. Clin. Invest.*, **90**, 1436–1442.

KAYE SB, LEWIS CR, PAUL J, DUNCAN ID, GORDON HK, KITCHENER HC, CRUICKSHANK DJ, ATKINSON RJ, SOUKOP M, RANKIN EM, CASSIDY J, DAVIS JA, REED NS, CRAWFORD SM, MACLEAN A, SWAPP GA, SARKAR TK, KENNEDY JH AND SYMONDS RP. (1992). Randomised study of two doses of cisplatin with cyclophosphamide in epithelial ovarian cancer. *Lancet*, **340**, 329–333.

KRAUSE CJ, CAREY TE, OTT RW, HURBIS C, CLATCHEY KD AND REGEZI JA. (1981). Human squamous cell carcinoma: establishment and characterization of new permanent cell lines. *Arch. Otolaryngol.*, **107**, 703–710.

KWOK TT AND SUTHERLAND RM. (1989). Enhancement of sensitivity of human squamous carcinoma cells to radiation by epidermal growth factor. *J. Natl Cancer Inst.*, **81**, 1020–1024.

LIEBER M, SMITH B, SZAKAL A, NELSON-REES W AND TODARO G. (1976). A continuous tumor cell line from a human lung carcinoma with properties of type I alveolar epithelial cells. *Int. J. Cancer*, **17**, 62–70.

MANN SC, ANDREWS PA AND HOWELL SB. (1991). Modulation of cis-diamminedichloroplatinum (II) accumulation and sensitivity by forskolin and 3-isobutyl-1-methylxanthine in sensitive and resistant human ovarian carcinoma cells. *Int. J. Cancer*, **48**, 866–872.

MICKEY DD. (1977). Heterotransplantation of a human prostatic adenocarcinoma cell line in nude mice. *Cancer Res.*, **37**, 4049–4058.

NAKATA B, BARTON R, HOWELL SB AND LOS G. (1994). Association between over-expression of hsp60 and cisplatin resistance in head and neck cancer cells. *Proc. Am. Assoc. Cancer Res.*, **35**, 466.

SAGGI SJ, SAFIRSTEIN R AND PRICE RM. (1992). Cloning and sequencing of the rat preproepidermal growth factor cDNA: comparison with mouse and human sequences. *DNA Cell Biol.*, **11**, 481–487.

SEMPLE TU, QUINN LA, WOODS LK AND MOORE GE. (1978). Tumor and lymphoid cell lines from a patient with carcinoma of the colon for a cytotoxicity model. *Cancer Res.*, **38**, 1345–1355.

TAETLE R, JONES O, HONEYSETT J, ABRAMSON I, BRADSHAW C AND REID S. (1987). Characterization of xenograft-derived melanoma cell lines. *Cancer*, **60**, 1836–1841.

WILSON AP, FORD CHJ, NEWMAN CE AND HOWELL A. (1987). Cisplatinum and ovarian carcinoma. *In vitro* chemosensitivity of cultured tumor cells from patients receiving high-dose cisplatinum. *Br. J. Cancer*, **56**, 763–773.

WOLF CR, HAYWARD IP, LAWRIE SS, BUCKTON K, McINTYRE MA, ADAMS DJ, LEWIS AD, SCOTT AR AND SMYTH JF. (1987). Cellular heterogeneity and drug resistance in two ovarian adenocarcinoma cell lines derived from a single patient. *Int. J. Cancer*, **39**, 695–702.

YUNIS AA, ARIMURA GK AND RUSSIN DJ. (1977). Human pancreatic carcinoma (MIA PaCa-2) in continuous culture: sensitivity to asparaginase. *Int. J. Cancer*, **19**, 128–135.

ZIJLSTRA JG, DE VRIES EGE AND MULDER NH. (1987). Multifactorial drug resistance in an adriamycin-resistant human small cell lung cancer cell line. *Cancer Res.* **47**, 1780–1784.